# The Evolution of Dieulafoy's Lesion Since 1897: Then and Now—A Journey through the Lens of a Pediatric Lesion with Literature Review

**DOI:** 10.1155/2012/432517

**Published:** 2012-02-14

**Authors:** Jenna-Lynn Senger, Rani Kanthan

**Affiliations:** Department of Pathology and Laboratory Medicine, Royal University Hospital, University of Saskatchewan, Saskatoon SK, Canada S7N 0W8

## Abstract

*Background*. In 1897, Dieulafoy was the first to characterize a gaping arteriole within the gastric mucosa causing massive hematemesis, designating it as “exulceratio simplex.” A hundred years later, this vascular abnormality, now commonly referred to as a “Dieulafoy lesion,” has been identified through the entire gastrointestinal tract and the bronchus. *Objectives and Methods*. As the original findings have been subjected to revisions and modifications by modern authors, Dieulafoy's original paper was reviewed and analyzed. The evolution of the current usage of “Dieulafoy's lesion” in the literature has been summarized with comparisons to the original report. Additionally, an index case of a 10-year-old female with a gastric “exulceratio simplex” is reported with a review of previously reported paediatric Dieulafoy lesions. *Conclusions*. The term “Dieulafoy lesion” in modern literature no longer adheres to the initial conclusions with regards to its origin, demographics, and presenting symptoms. Dieulafoy lesions remain a rare cause of gastrointestinal bleeding that can cause life-threatening haemorrhages in children.

## 1. Introduction

Paul Georges Dieulafoy (1839–1911), a professor of pathology at the Faculty of Medicine in Paris, France, was the first to describe a series of 10 patients who presented with massive hematemesis (approximately 4 L of blood in less than 24 hours) due to a bleeding gastric vessel, without any evidence of ulceration in the first three lessons of the 1897-1898 edition of “Clinique Médicale de l‘Hôtel-Dieu.” At autopsy, a superficial ulceration with a gaping arteriole was found in the gastric submucosa. The lesion's borders were not hardened or projecting, and the remainder of the mucosa was in perfect health. Dieulafoy concluded that this lesion was not a typical gastric ulcer and named it an “exulceratio simplex,” which in time became known as a “Dieulafoy lesion” [1].

Modern literature has broadened the original definition of a “Dieulafoy lesion.” Initially only described in the stomach, such findings have been additionally reported in the rest of the gastrointestinal tract such as the esophagus [2], duodenum [3], ileum [4], jejunum [5], colon [6], anal canal [7], and rectum [7, 8]. Dieulafoy lesions have also been reported as a case series in nongastrointestinal sites such as the bronchus [9]. The advent of endoscopy has drastically changed the process of diagnosing and treating Dieulafoy lesions, with techniques such as endoscopic laparoscopic banding (ELB), thermocoagulation, and injections with adrenaline/epinephrine (EPI) as alternatives to replace surgical management as was described in the mid-1800s as best-practice management.

## 2. Materials and Methods

### 2.1. Objective

Dieulafoy's original treatise published in French was reviewed, analyzed, and translated into English for discussion as we suspected that the original findings have been subjected to revision with modification by many current authors. Using an index case of a 10-year-old female who presented with recurrent upper gastrointestinal bleeding that necessitated a partial gastrectomy, the pathological abnormality of the vascular anomaly in the submucosa—“Dieulafoy lesion”—is analyzed from a historical viewpoint of “then and now.” All published paediatric cases of Dieulafoy lesions as available in PubMed and Medline have also been retrieved and analyzed providing a comprehensive review of paediatric Dieulafoy lesions.

**Part 1: Revisiting the Past**

We obtained a copy of the original first three lessons of “Clinique Médicale de l‘Hôtel-Dieu” treatise written in 1897-1898 by Paul Georges Dieulafoy. The original French paper was read and analyzed in its native language and translated into English for detailed review and study.

**Part 2: Analyzing the Present**

The definition and usage of a “Dieulafoy lesion” in modern literature was explored. The most widely recognized definition of a “Dieulafoy Lesion” was retrieved from Wikipedia, an extensively accessed free online encyclopaedia. We then researched the definition, epidemiology/aetiology, clinical presentation, detection, and treatment of this lesion using Google Scholar, PubMed, and Medline. Finally, a search of PubMed and Medline limited to the paediatric population (ages 0–18) was conducted in the English and French languages. All references were read, reviewed, and analyzed.

**Part 3: Moving Forward: a New Paediatric Index Case**

A 10-year-old female presented to the Emergency Room with melena, hematemesis, and generalized abdominal pain. At presentation, her haemoglobin was 6.5 g/dL with a blood pressure of 99/64. Gastroscopy identified four polypoid lesions on the lesser curvature surrounded by an erythematous and friable mucosa that was oozing blood. Multiple mucosal biopsies showed no significant pathological abnormality. Imaging including an MRI showed no significant findings, and a repeat gastroscopy was unremarkable. She was discharged home on prevacid.

A year and half later, she was admitted to the paediatric intensive care unit (PICU) after vomiting approximately 1 L of bright red blood preceded by a one-day history of general malaise and fatigue. She was hypotensive at presentation, with a systolic pressure of 70 mmHg and haemoglobin of 7.5 g/dL. She was stabilized by transfusion with fluids and two units of packed red blood cells (PRBCs). The abdominal ultrasound was normal. A repeat gastroscopy rediscovered the 4-5 cm region of erythematous and friable gastric mucosa and raised the possibility of an underlying vascular abnormality. A second repeat MRI revealed diffuse thickening of the greater curvature with several enlarged mesenteric lymph nodes; the largest measured 5-6 mm.

**Figure 1:**
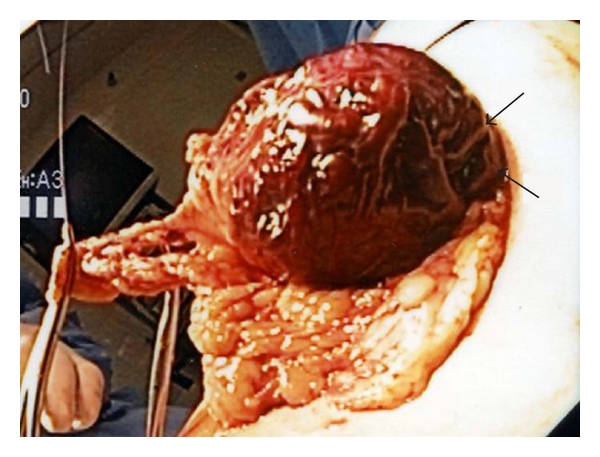
Intraoperative specimen of stomach at partial gastrectomy showing the rich vascular abnormality seen within the posterior gastric wall (arrow).

On laparoscopic examination, prominent, tortuous vessels were seen on the lateral aspect of the greater curvature and extensively on the posterior wall of the stomach. The greater omentum inhibited a full visualization, and it was decided to carry out an exploratory laparotomy to determine the full extent of this vascular abnormality. At intraoperative examination, mild handling of the stomach caused extensive bleeding, which required vascular clamps and two units of PRBCs to maintain haemostasis. No “normal” gastric wall was visible on the posterior aspect (Figure 1) resulting in the decision to proceed with a partial gastrectomy to include the anterolateral and posterior walls of the stomach.

**Figure 2:**
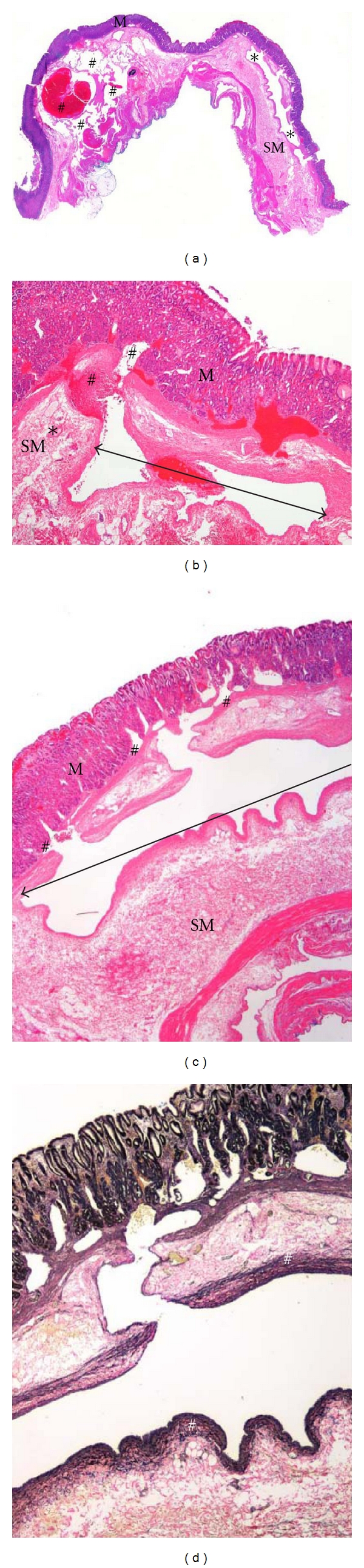
Histopathological findings. (a) Whole-mount scan of hematoxylin and eosin stained slide showing the exulceratio simplex (∗) in the submucosa (SM) (below the mucosa (M)) as described by Dieulafoy. Additionally, (#) shows the presence of a complex rich anastomosis of varying sized vessels in the submucosa with an intraluminal haemorrhagic thrombus (cirsoid aneurysm of gastric vessels). (b) Low magnification (objective lens ×2) of hematoxylin and eosin-stained slides shows the presence of blood within the large tortuous, caliber-persistent artery () in the submucosa (SM) with evidence of rupture and haemorrhage (#) through the overlying mucosa (M). A normal-sized submucosal arteriole (∗) is seen at the same level. (c) High magnification (objective lens ×10) of hematoxylin and eosin-stained slides shows the presence of the large caliber-persistent artery () in the submucosa (SM)—Dieulafoy's lesion—with communications (#) into the overlying noninflamed mucosa (M). (d) High magnification (objective lens ×10) of elastin-stained slides shows the presence of elastin in the wall (#) of the exulceratio simplex confirming its arterial origin.

Histopathological examination of the specimen showed a hemorrhagic area measuring 3.3 × 2.2 cm with a central 0.4 × 0.4 cm vascular polyp. On microscopic examination, a large dilated tortuous vessel with evidence of fibrin thrombi and recent haemorrhage through the overlying mucosa was identified (Figures 2(a) and 2(b)). This submucosal vessel measured up to 8.3 mm in some sections. No accompanying vein was identified. The mucosa appeared congested with pseudopolypoid regions, and there was no evidence of inflammation or significant erosion. Special stain demonstrated the presence of elastin in the wall of the persistent large vessel confirming its arterial origin (Figures 2(c) and 2(d)). Additional sections also showed the presence of complex anastomosing submucosal vessels with mucosal and submucosal evidence of interstitial haemorrhage. The diagnosis of a Dieulafoy's lesion in the proximal stomach was confirmed.

Postoperative recovery was uneventful. As she had no further complaints at her followup at the outpatient clinic 6 weeks later, her care has now been transferred to her family physician.

All information provided in this paper has been completely deidentified and is thus exempted from a formal review by the Ethics Committee as per our institutional policy.

## 3. Results and Discussion

### 3.1. Then: The Original Dieulafoy Lesion

Though in 1884 Gallard wrote the first description of a patient with a “Dieulafoy's lesion” [10], this eponym is attributed to the publication of Clinique Médicale de l‘Hôtel-Dieu de Paris written by Paul Georges Dieulafoy. He was the first to characterize in detail a lesion he called “exulceratio simplex.” In the first three lessons of this treatise, Dieulafoy describes 10 cases of exulceratios and notes that the features that differentiate these lesions from ulcers are caused by tuberculosis, syphilis, typhoid fever, uremia, and alcoholism. Histological examination of the lesion revealed “*sur l'une des petites érosions cratériformes, due à la disparition de la muscularis mucosae, apparaît làrtériole béante” *(“on one of the small crateriform erosions, caused by disappearance of the muscularis mucosa, appears a gaping arteriole,” page 4). He concludes the three lessons with eight major learning points about the “exulceratio simplex” (pages 42-43) [1]:

1. the exulceratio simplex is *“à l'estomac, une perte de substance très superficielle et assez étendue” *(“in the stomach, a loss of substance very superficial and fairly extensive,” Page 42);
2. the “exulceratio simplex” is oval, elliptical, or star shaped and has the dimensions of a 50-centime piece, of 1 franc or 2 francs. It is *“très superficielle et ne dépasse pas en profondeur la tunique muqueuse”* (“very superficial and does not extend deeper than the tunica mucosa," page 42);
3. edges of the exulceratio “*n'étant ni indurés ni surelevés*” (“neither indurate nor raised,” page 42) and the gastric walls maintain their softness;
4. the exulceratio “*est un processus ulcéreux aigu*” (“is an acute ulcerative process,” page 43) that reaches a voluminous and superficial submucosal arteriole;
5. rarely the exulceratio simplex presents with gastric symptoms, but more often it evolves *“silencieusement, sournoisement” *(“silently, slyly,” page 43) in a healthy patient who may develop nausea, vertigo, syncope, and large haematemeses;
6. to diagnose exulceratio simplex, the physician must identify a “true” haematemesis and then *“acquérir la certitude que la lesion … siège bien à l'estomac” *(“gain certainty that the lesion is sitting in the stomach,” Page 43);
7. the exulceratio simplex is extremely serious due primarily to the substantial haematemeses;
8. the future risk of recurrence remains unknown.

### 3.2. Now: Dieulafoy Lesions Described in the Current Literature

In the current literature, alternative names, descriptions, and criteria for Dieulafoy lesions include “caliber-persistent artery,” “gastric aneurysm,” “gastric arteriosclerosis,” “submucosal arteriole malformation,” and “cirsoid aneurysm” [11, 12]. The “exulceratio simplex” as described in 1897 is estimated to account for 0.5–14% of upper GI bleeding [13, 14]. It is, however, suggested that a lack of recognition rather than true rarity may be responsible for this low incidence [15]. Most lesions (75–95%) are in the proximal stomach, particularly within 6 cm of the gastroesophageal junction on the lesser curve [16], yet they have also been described in nongastric sites such as the duodenum [3], jejunum [5], ileum [17], rectum [8], and in nongastrointestinal sites such as the bronchus [9]. This clearly digresses from Dieulafoy's sixth learning point which states that the lesion “*siège bien à lèstomac*” (“sits in the stomach,” page 43) [1]. Dieulafoy lesions are more common in men than women, at a 2 : 1 ratio [18] and the mean age at presentation is within the 5th decade of life (range 50–70 years) [15]. This contrasts strongly with Dieulafoy's observation that “*l'exulceratio à une predilection pour les jeunes gens” *(“the exulceratio has a predilection for young people,” page 36) [1].

Dieulafoy lesions classically present as intermittent and massive GI haemorrhage with symptoms associated with this blood loss. 44% of patients report melena, 30% haematemesis, 18% with both hematemesis and melena, 6% with haematochezia, and 1% with iron-deficiency anemia [17] with the mean reported haemoglobin at admission ranging between 8.4 and 9.2 g/dL [13]. In Dieulafoy's original report, all patients presented with acute massive haematemesis such that his learning point no. 6 emphasizes the importance of accurate identification of haematemesis. He also stresses that *“les hémorragies stomacales dues à léxulceratio simplex sont … pas annoncées par de petits vomissements de sang*” (“gastric haemorrhages due to the exulceratio are not announced by little vomits of blood,” page 32) [1]. In our index case, however, the acute massive bleed was preceded by “little vomits of blood” that occurred almost a year and a half prior. Perhaps due to the older age of onset, comorbidities are present in up to 90% of patients, the most common including ischemic heart disease, hypertension, diabetes mellitus, liver disease, and renal failure [12]. Interestingly Dieulafoy commented that patients with the “exulceratio simplex” “*ressemblent à des gens bien portants*” (“seemed like healthy people,” page 41 and conclusion no. 5) [1].

Though over a hundred years have passed since Dieulafoy first described this lesion, the mechanisms causing the tortuosity and the persistence of the large-sized submucosal arteries remain unknown. Many reports have attributed this lesion to the pathological structure or the size of the artery with a persistent musculoelastic mantle of Wanke. Debate continues regarding the exact pathology and linkage of this artery to the overlying mucosa [35]. Further, what actually triggers their submucosal-to-mucosal rupture has yet to be proven. No family predisposition [3] or risk factors have been identified despite research that has searched for a link with nonsteroid anti-inflammatory drugs (NSAIDs), alcohol, tobacco, or a history of peptic ulcer disease [7]. The lesion's predominance on the lesser curve of the stomach may be explained by the vascular architecture of this region which arises directly from an arterial chain [15]. The most widely accepted theory which takes into account the paediatric cases suggests that the tortuous artery with an increased diameter and often a variable course length is a congenital anomaly [25]. As such, a large submucosal artery may be abnormally close to the mucosa, facilitating its emergence and making it more vulnerable to rupture [32]. All patients of the original series had only a single ruptured artery, leading Dieulafoy to note that “*elle passerait assez facilement inaperçue sans un examen attentive et sans l'idée préconcue qu'on va la trouver” *(“it would easily enough pass unperceived without a thorough examination and without the preconceived idea that we will find it,” page 24) [1]. The theories of the cause of rupture are more variable, but it is agreed that something causing mucosal erosion or ischemic injury must be the initial step [17]. The three principal theories of pathogenesis include the following: (a) pulsations of the abnormally large artery may disrupt the mucosa and exposure of the artery to gastric/bowel contents which then chemically or mechanically erode and cause the bleed [12, 17], (b) gastric “wear and tear” promotes the formation of an arterial thrombus that causes necrosis [17], and (c) age-related atrophy [17].

The advent of endoscopy has marked a drastic change in the diagnostic evaluation and therapeutic management of Dieulafoy lesions. Up to 71% of initial endoscopies are diagnostic only if there is active haemorrhage of at least 0.5 mL/min [15]. However, in 6% of patients, the intermittent nature of the bleeding often requires multiple endoscopies to establish a definitive diagnosis [12, 17]. Unsuccessful endoscopies in 44% of patients are due to the presence of excess of blood in the stomach/bowel, and the remaining 56% are subtle lesions that are often overlooked [12, 17]. Because of this subtlety, concomitant lesions such as ulcers or varices may be incorrectly diagnosed as being responsible for the bleed [13]. To diagnose a Dieulafoy lesion, the endoscopic visual criteria that must be met include (a) active arterial spurting or oozing from a small (<3 mm) defect in the mucosa, (b) visualization of a vessel protruding from a slight defect or normal mucosa, and/or (c) a fresh blood clot adherent to a defect of normal mucosa as described by Dieulafoy [1, 16].

Should endoscopy fail to determine a source of bleeding, angiography and capsule endoscopy may provide useful information. On an angiogram, extravasation of contrast into the GI system from an eroded artery is indicative of this lesion, as is the presence of tortuous ectatic vessels in the arterial phase without early venous return thereby distinguishing this unique lesion from arteriovenous malformation [12]. This diagnostic modality is particularly useful for colorectal lesions where poor bowel preparation may obscure colonoscopy results [17]. A relatively new option, capsule endoscopy, is a noninvasive, direct way of investigating areas of the GI tract that are difficult to access by EGD or colonoscopy. There is a risk, however, of missing these small lesions if the camera is pointed to the wrong way and is of limited use in an emergency situation [17]. There is limited knowledge about the use of capsule endoscopy in children, as its size precludes its usefulness [17, 24].

Since 1986, endoscopy in addition to being the primary diagnostic tool has also become the first-line method of treatment [25]. Traditionally treated with a gastrotomy or gastrectomy, surgery is now reserved for the 4–8% of cases that do not achieve endoscopic haemostasis [36]. The three main forms of endoscopic treatment include (a) thermal electrocoagulation, heat probe coagulation, argon plasma coagulation, (b) regional injection-epinephrine (EPI) or norepinephrine (NOR) injection and sclerotherapy, and (c) mechanical banding and hemoclips [17, 32, 36]. A recent study by Alis et al. found that endoscopic band ligation (EBL) was associated with a significantly lower risk of recurrent bleeding as well as a shorter hospital stay when compared with sclerotherapy [36]. Monotherapy has a 9–40% higher risk of rebleeding versus combination therapy [17]. The site of the bleed is often tattooed with India ink for future identification in the event of a recurrent bleed [18]. The overall mortality rate of Dieulafoy lesions is 8.6%, which is constant whether treated endoscopically or surgically [12]. The mainstay of surgical treatment for endoscopic failures is wide wedge resection or local excision such as partial/wedge gastrectomy [37]. Simple oversewing of the lesion is not recommended as it is associated with a greater risk of recurrent bleeding [37].

### 3.3. Pediatric Dieulafoy Lesions

Dieulafoy lesions as a cause of massive gastrointestinal bleeding are uncommon occurrences in the paediatric population. The most common cause of massive GI bleeding in children is usually related to oesophageal varices secondary to portal hypertension and chronic liver disease [27, 30]. These “exulceratio simplex” are infrequent in children, and in his original description, Dieulafoy did not encounter any children with this rare form of gastrointestinal bleeding [1].

As listed in Table 1, twenty four cases of paediatric Dieulafoy lesions have been published in the English language as available in PubMed and Medline between 1990 and 2011 [4, 6, 7, 15, 19–34, 38–41]. Though the reported age ranged from 8 weeks [34] to 18 years [25, 32, 33], children tended to be older, with the mean age of 10 years.

Unlike Dieulafoy lesions in the adult population, there is no clear preponderance of these lesions in pediatric males. Such strong male predominance in adults is perhaps hormonally related and can be attributed to the lack of estrogenic protective effects in males. It is also postulated that complex hormonal dysfunctions at the submucosal arteriole arcade are involved in the pathogenesis of the caliber-persistent artery as described by Dieulafoy's illustrations on pages 15 and 16 [1].

The location of Dieulafoy lesions reported in children includes ten in the stomach, five in the jejunum, five in the ileum, and two in each the duodenum, and rectum and one in each of the sigmoid, anorectal junction, and anal canal. Twelve children (46%) were treated surgically, a much higher percentage than in the adult population already described, while 14 were managed endoscopically by injection therapy, band ligation, and thermocoagulation (Table 1). Recently, minimally invasive diagnostic and therapeutic techniques such as capsule endoscopy assisted by laparoscopic resections, conventional bidirectional endoscopy, push enteroscopy, and radionuclide imaging with techniques such as multidetector-row computed tomography (MDCT) have opened new avenues in the management of obscure and occult gastrointestinal bleeding in children [8, 24]. Further, GI bleeding in children continues to pose many different challenges to the clinician as there is no general consensus in the investigative and treatment protocols to be followed [24].

## 4. Conclusion

The usage of the term “Dieulafoy lesion” in the current literature no longer fits within the confines of the conclusions drawn by its namesake over a hundred years ago. Dissidents contest with Dieulafoy's original theory that the “exulceratio simplex” though of congenital origin represents an acute process. Other digressions now include an aging population, nongastric and extraintestinal locations, and its recognition in children as well as in adults. Though massive haematemesis—“gastrorrhagias”—was the sine qua non of the “original” Dieulafoy lesion, the inclusion of nongastric (oesophagus, duodenum, jejunum, ileum, colon, anal canal, and rectum) and extraintestinal (bronchus) sites of this anomaly has completely altered the clinical presentation. Additionally, endoscopic vascular criteria have replaced the diagnosis of this lesion in these widespread locations.

Nevertheless, the name “Dieulafoy lesion” is entrenched in the literature and contains historical significance; therefore, we suggest that this eponym may still be used with the understanding that the diversity of the lesion it describes no longer conforms to the original definition of the term. Dieulafoy lesions remain a rare and most dangerous form of gastrointestinal bleeding that can result in life-threatening haemorrhages in children.

## Figures and Tables

**Table 1:** Paediatric Dieulafoy lesions 1990–present (PubMed and Medline search “Dieulafoy” limited to paediatrics (0–18 years) and English/French languages.

Ref no.	Author		Age (Sex)	Presentation	Hb at presentation	Location of Dieulafoy lesion	Treatment
[19]	Shibutani et al. 2011	Case report	14 (F)	Loss of consciousness, massive hematochezia	8.4 g/dL	Ileum	Laparotomy, resection
[20]	Itani et al. 2010	Case report	6 (F)	Painless blood per rectum	13.9 g/dL	Sigmoid	Endoscopic resection with cauterization
[21]	Ezzat et al. 2010	Case report	7 (F)	Fever, abdominal pain, nausea, vomiting, and diarrhea	7.1 g/dL	Terminal ileum	Explorative laparotomy, ileocolectomy
[22]	Moreira-Pinto et al. 2009	Case report	14 (F)	Nausea, vomiting, dizziness, and loss of consciousness	10 g/dL	Jejunum	Segmental enterectomy
[23]	Marangoni et al. 2009	Case report	15 (F)	Melena, hematemesis, tachycardia, and hypotensive	NR	(1) Greater curvature stomach, (2) jejunum	(1) Injection NOR, endoscopic clip, and anterior gastrectomy; (2) small bowel resection
[24]	Prasad et al. 2007	Case report	13 (M)	Giddy, hypotensive	7.5 g/dL	Jejunum	Laparoscopic-assisted wedge resection jejunum
[25]	Linhares et al. 2006	Series	18 (NR)	Upper gastrointestinal bleeding	NR	Gastric lesion	Endoscopic sclerotherapy
[26]	Valera et al. 2006	Series	16 (M)	Gastrointestinal haemorrhage	NR	Fundus of stomach	Endoscopic injection therapy and band ligation
[27]	Avlan et al. 2005	Case report	3 (M)	Bloody vomiting, epigastric pain, and	8.6 g/dL	Corpus stomach	Endoscopic injection of EPI
[28]	Morowitz et al. 2004	Case report	4 (M)	lethargy abdominal pain, hematochezia, and blood per rectum	8.5 g/d:	Ileum	Laparoscopic segmental resection of ileum
[29]	Lilje et al. 2004	Case report	13 mo (M)	Haemorrhagic shock, hematemesis, melena, fatigue, and fever intermittent vomiting	6.3 g/dL	Stomach	Endoscopic injection of EPI
[30]	Pitcher et al. 2002	Case report	5 (M)	Massive hematemesis, haemorrhagic shock	3 g/dL	Incisura of stomach	Laparotomy with oversewing of the lesion
[31]	Owaki et al. 2002	Case report	12 (F)	Gastrointestinal bleed	NR	Distal jejunum	Partial jejunectomy
[4]	Shibutani et al. 2001	Case report	14 (F)	Massive hematochezia, tachycardia, and hypotensive	8.4 g/dL	Ileum	Laparotomy-ileal resection with primary anastomosis
[32]	Blecker et al. 2001	Case report	18 (M)	Syncope, sense of fullness, maroon-colored stool	8.1 g/dL	Proximal jejunum	Laparotomy resection of jejunal lesion
[33]	Guy et al. 2001	Case report	18 (F)	Painless rectal bleeding	5 g/dL	Anorectal junction	Surgical
[34]	Stockwell et al. 2000	Case report	8 week (F)	Respiratory distress, bloody oral secretion, pallor, and responsive only to painful stimuli	9.6 g/dL	Stomach	Sclerotherapy
[7]	Meister et al. 1998	2 case reports	5 (F)	Painless rectal bleeding	NR	Rectum	Injection with EPI and thermocoagulation
